# Multi-Objective Trajectory Optimization of a 7-DOF Wiring Robot for Aircraft Harness Layout on Fixture Boards

**DOI:** 10.3390/s26185714

**Published:** 2026-09-09

**Authors:** Jinhua Cai, Tao Jiang, Han Hou, Peng Gao, Na Liu

**Affiliations:** 1 School of Mechatronic Engineering, Changchun University of Science and Technology, No. 7089, Satellite Road, Chaoyang District, Changchun 130012, China; 2023200111@mails.cust.edu.cn; 2 Chongqing Research Institute, Changchun University of Science and Technology, No. 5, Yulin Road, Liangjiang New Area, Chongqing 401120, China; 3 High-End Aeronautical Equipment Technology Innovation Center (Sichuan) Co., Ltd., Chengdu 610000, China

**Keywords:** aircraft wire harness, 7-DOF robot, quintic B-spline, multi-objective trajectory optimization, constraint handling, DE-NSGA-II, digital twin

## Abstract

Aircraft wire-harness routing on fixture boards requires not only efficient large-workspace robot motion but also smooth and process-safe passage around densely distributed pins. Conventional robot trajectory optimization generally focuses on generic motion indices and cannot explicitly represent local over-bending risk and coupled wiring-process constraints. This paper proposes a process-safety-aware multi-objective trajectory optimization method for a seven-axis wiring robot composed of a linear rail and a six-DOF manipulator. An executable reference trajectory is parameterized using quintic B-splines, and execution time, normalized joint jerk, and a pin-neighborhood weighted bending-safety proxy are jointly optimized under robot-motion, pin-passing, board-clearance, and non-target-pin constraints. A constraint-guided adaptive DE-NSGA-II algorithm is developed to improve feasible-solution search in the resulting narrow and strongly coupled feasible region. Physical experiments show that, compared with MOPSO, the proposed method reduces normalized joint jerk by 35.8% and the bending-safety proxy by 77.0%, while completing all ten repeated trials without controller alarms. Vision-based measurements further show a strong correlation between the proposed proxy and the actual harness bending radius ($R^{2}=0.991$). Additional routing layouts confirm the applicability of the method to more complex fixture-board configurations. These results demonstrate that the proposed method provides a practical balance among routing efficiency, trajectory smoothness, and process reliability.

## 1. Introduction

Aircraft wire harnesses are key carriers for power transmission, signal exchange, and airborne-system interconnection. Their manufacturing quality directly affects aircraft reliability, safety, and maintainability. Compared with ordinary electrical products, aircraft harnesses usually have many branches, complex topologies, long routing spans, small batches, and strict process-consistency requirements. Their layout on two-dimensional fixture boards is still largely performed with pin arrays and manual routing. Recent reviews show that wire-harness manufacturing remains one of the least automated operations in automotive, aerospace, and complex-equipment manufacturing. The main barriers are the manipulation of deformable linear objects, configuration variability, uncertainty in perception and grasping, and the difficulty of simultaneously achieving flexibility and efficiency [1,2,3,4]. Therefore, trajectory optimization for robotic fixture-board wiring is needed to improve routing efficiency and trajectory repeatability while satisfying process-safety constraints.

Robotic wire-harness manufacturing has been investigated from the perspectives of visual perception, dual-arm collaboration, CAD-driven programming, and quality inspection. Nguyen and Yoon proposed a vision-based method for extracting the three-dimensional profile of a wire harness in robotic assembly [5]. Nguyen et al. further developed a 3D vision method for detecting multiple wire branches, providing a perception basis for grasping and routing [6]. Cho et al. proposed a dual-arm cable-wiring strategy that integrates cable-state estimation, friction modeling, compliance control, and trajectory planning [7]. González Huarte et al. developed a CAD-based robotic programming solution for aeronautical wire-harness manufacturing, in which fixture-board and harness-design data are used to generate initial routing trajectories [8]. Song et al. presented an artificial-intelligence-based fault detection system for wiring-harness manufacturing [9]. In addition, Malvido Fresnillo et al. built a dual-arm robotic system for automated multi-branch wire-harness assembly [10]; Liu et al. investigated the design and motion planning of a 7-DOF assembly robot for spacecraft modules [11]; and Jiang et al. demonstrated the feasibility of robotic wire-harness assembly in an automotive production line [12]. These studies indicate a transition from isolated perception or local manipulation toward system-level robotic integration. However, trajectory optimization for a large aircraft fixture-board system composed of a linear external axis and a six-DOF manipulator remains insufficiently studied. Overall, existing wire-harness robotic studies mainly focus on perception, manipulation, offline programming, or inspection, whereas the systematic integration of large workspace coverage, trajectory smoothness, and wire-specific process safety remains limited.

Aircraft harness layout is not a conventional rigid-part handling task. It is a process operation involving deformable linear objects. Surveys on deformable-object manipulation have shown that cables, ropes, and harnesses have infinite-dimensional configurations, uncertain deformation, and strong contact coupling with the environment during grasping, guiding, contact, and shaping [13]. Tang et al. studied shape alignment of deformable linear objects using coherent point drift [14]. Zhu et al. considered environmental contacts in manipulation planning for deformable linear objects [15]. Almaghout et al. investigated robotic co-manipulation of deformable linear objects in large-deformation tasks [16]. Wakamatsu and Hirai established a static deformation model of linear objects based on differential geometry, providing a classical basis for cable-shape modeling [17]. Recent soft-robotic research, such as the origami flexiball-inspired soft robotic jellyfish, also shows that compliant deformation strongly affects robotic motion behavior [18]. This further supports the need to consider deformation-related safety in trajectory planning. In fixture-board wiring, the robot end-effector must follow a pin sequence and locally bypass pins while avoiding excessive bending near pins, abrupt pose changes, local rubbing, board interference, and collisions with non-target pins. Thus, end-effector tracking error or generic energy consumption alone cannot adequately represent the process-safety requirements of aircraft harness routing.

Robot trajectory planning commonly optimizes execution time, velocity, acceleration, jerk, energy consumption, and dynamic load. Wang et al. showed that jerk-bounded trajectories help reduce motion shock and improve real-time execution [19]. Huang et al. optimized time and jerk for industrial robots and demonstrated Pareto trade-offs between efficiency and smoothness [20]. B-splines, NURBS, and polynomial trajectories have also been widely used in joint-space planning [21,22,23,24,25,26,27,28,29]. Recent studies further extended robot motion optimization by integrating process constraints, motion-performance reliability, and positioning uncertainty. For example, trajectory optimization for a rigid-flexible coupling spray-painting robot incorporated coating-process constraints [30], while reliability-oriented studies analyzed robotic motion performance and manipulator positioning accuracy under uncertainty [31,32]. These works indicate that robot trajectory optimization should consider not only generic motion indexes but also task-specific constraints and reliability. However, existing studies mainly address rigid operations, spraying, machining, assembly, or general manipulator performance. They rarely incorporate wire-bending radius, pin-neighborhood risk, fixture-board clearance, and non-target-pin avoidance as coupled objectives and constraints for aircraft wire-harness routing.

Multi-objective evolutionary optimization provides effective tools for nonlinear, non-convex, and strongly constrained trajectory planning. The NSGA-II algorithm proposed by Deb et al. improves multi-objective search efficiency through fast non-dominated sorting and crowding-distance preservation [33]. Differential evolution, introduced by Storn and Price, provides strong global search ability for continuous variables [34]. MOPSO, proposed by Han, is also frequently used in complex engineering multi-objective optimization [35]. For constraint handling, Deb’s feasibility-priority rule provides a classical framework for constrained evolutionary optimization [36]. Zitzler et al. reviewed performance indicators such as hypervolume [37], and MOEA/D, epsilon-dominance-based multi-objective differential evolution, and IGD-type indicators provide important bases for evaluating convergence and diversity of Pareto sets [38,39,40]. Mezura-Montes and Coello emphasized that complex constrained optimization depends not only on search operators but also on the coordination between constraint handling and the search process [41]. Digital twins can further provide virtual verification and risk pre-checking for offline optimized robot trajectories. Fuller et al., Tao et al., and Zhang et al. summarized the enabling technologies, industrial applications, and decision-support value of digital twins [42,43,44]. For aircraft harness routing, a digital twin can check pin bypassing, board clearance, local curvature, and potential collision risks before physical trials.

Despite this progress, existing studies do not jointly address the execution-level refinement of long-span rail–manipulator trajectories, local over-bending risk near fixture-board pins, and the narrow feasible region created by coupled robot and wiring-process constraints. Conventional time-, energy-, or jerk-oriented optimization is therefore insufficient to fully represent the process-safety requirements of aircraft wire-harness routing.

This study focuses on refining an executable reference trajectory obtained from offline programming rather than generating the wiring route itself. The contributions are threefold: (1) a process-safety-aware formulation that combines execution time, normalized joint jerk, and a pin-neighborhood weighted bending-safety proxy with pin-passing, board-clearance, and non-target-pin constraints; (2) a constraint-guided adaptive DE-NSGA-II solver integrating control-point offset encoding, feasibility-rate-driven search, hierarchical repair, and feasibility-priority selection; and (3) multi-level validation through simulation, ablation analysis, digital-twin pre-checking, repeated physical execution, and vision-based bending measurements. The proposed framework therefore converts a geometrically executable reference route into a smoother and more process-reliable robot trajectory.

The remainder of this paper is organized as follows. Section 2 describes the wiring system, kinematic model, and optimization problem. Section 3 presents the quintic B-spline parameterization, objective functions, and constraints. Section 4 introduces the constraint-guided adaptive DE-NSGA-II algorithm. Section 5 reports the numerical, ablation, digital-twin, physical-platform, scalability, and vision-based validation results. Finally, Section 6 summarizes the main findings and future work.

## 2. System Description and Problem Formulation

### 2.1. Aircraft Harness Layout on a Fixture Board

**Figure 1:**
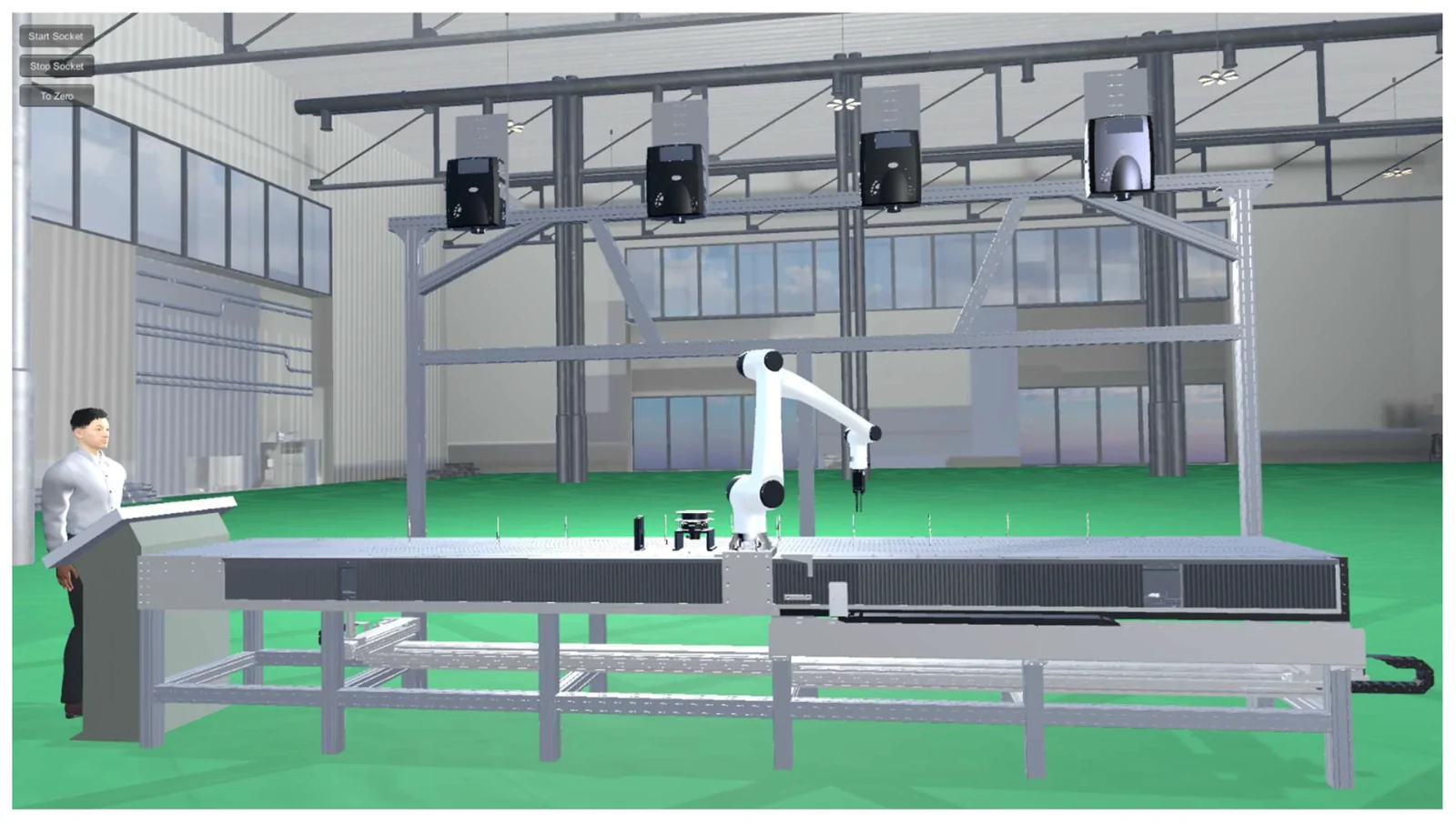
Schematic of the seven-axis wiring robot and aircraft harness fixture-board system.

The target task is the layout of an aircraft wire harness on a two-dimensional fixture board. A typical system consists of a linear rail, a six-DOF collaborative manipulator, a harness gripping/guiding end effector, a fixture board, a pin array, and a control system, as shown in Figure 1. The fixture board is approximately 5 m long. Pins or turning posts are mounted on its surface according to the process drawing to constrain the two-dimensional harness geometry. In aircraft harness manufacturing, the three-dimensional harness route is usually unfolded and mapped onto the fixture-board plane, forming a routing task defined by a pin sequence.

**Figure 2:**
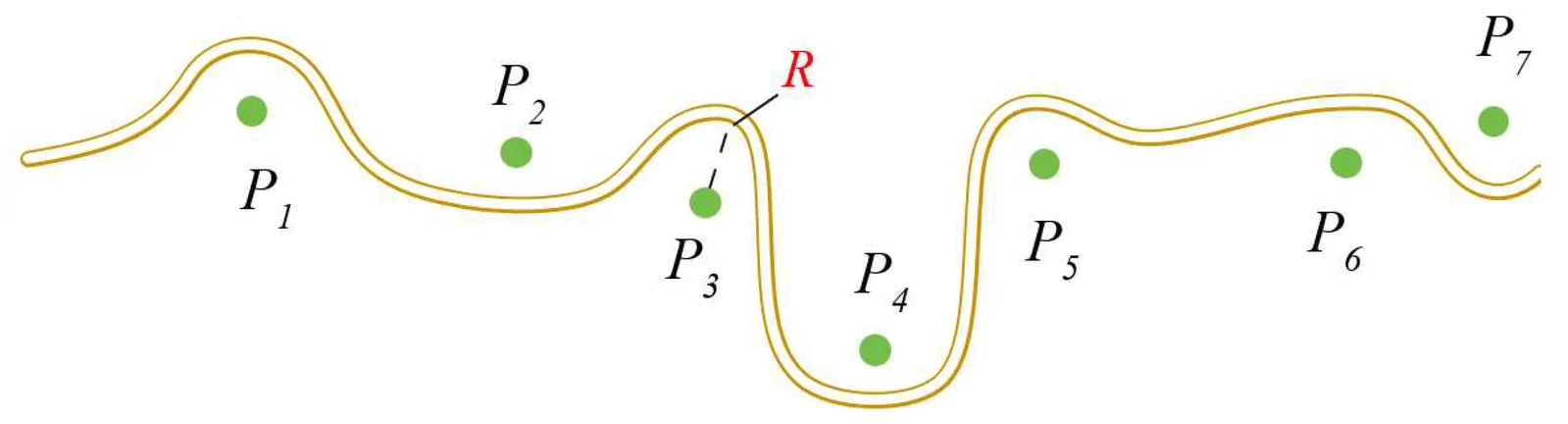
Local schematic of end-effector routing around fixture-board pins.

Let the target pin sequence on the fixture board be

(1) $$P=\{P_{1},P_{2},\ldots ,P_{N_{p}}\},\;P_{k}={\left[x_{k},y_{k},z_{k}\right]}^{T},\;k=1,2,\ldots ,N_{p}$$

where $N_{p}$ is the number of pins and $P_{k}$ is the target passing point or bypassing reference position of the kth pin. During routing, one end of the harness is fixed near the starting pin, while the robot end effector grips or guides the other end and passes through the pin neighborhoods in the specified order. The final harness shape must conform to the process drawing. Unlike rigid-part transfer, harness routing is a deformable-linear-object operation. The end-effector trajectory must satisfy robot motion constraints and must also avoid excessive local bending, rubbing, board interference, and collisions with non-target pins. Figure 2 illustrates the local routing path around pins. The green circles denote pins, the yellow curve denotes the end-effector guiding trajectory, and the local bending radius when passing near $P_{3}$ is denoted by R.

To focus the study, the front-end process-path parsing, pin-order determination, and initial inverse-kinematics solution are regarded as known inputs. This work focuses on the coordinated optimization of execution time, smoothness, and bending safety based on an executable reference trajectory. This treatment is consistent with the offline programming workflow used in small-batch and multi-variety aircraft harness production and allows the optimized trajectory to be imported into a digital twin and a physical controller for validation.

### 2.2. Kinematic Model of the Seven-Axis Wiring Robot

The seven-axis wiring robot consists of a linear-rail external axis and a six-DOF manipulator. The joint vector is defined as

(2) $$q\left(t\right)={\left[q_{0}\left(t\right),q_{1}\left(t\right),q_{2}\left(t\right),q_{3}\left(t\right),q_{4}\left(t\right),q_{5}\left(t\right),q_{6}\left(t\right)\right]}^{T}$$

**Figure 3:**
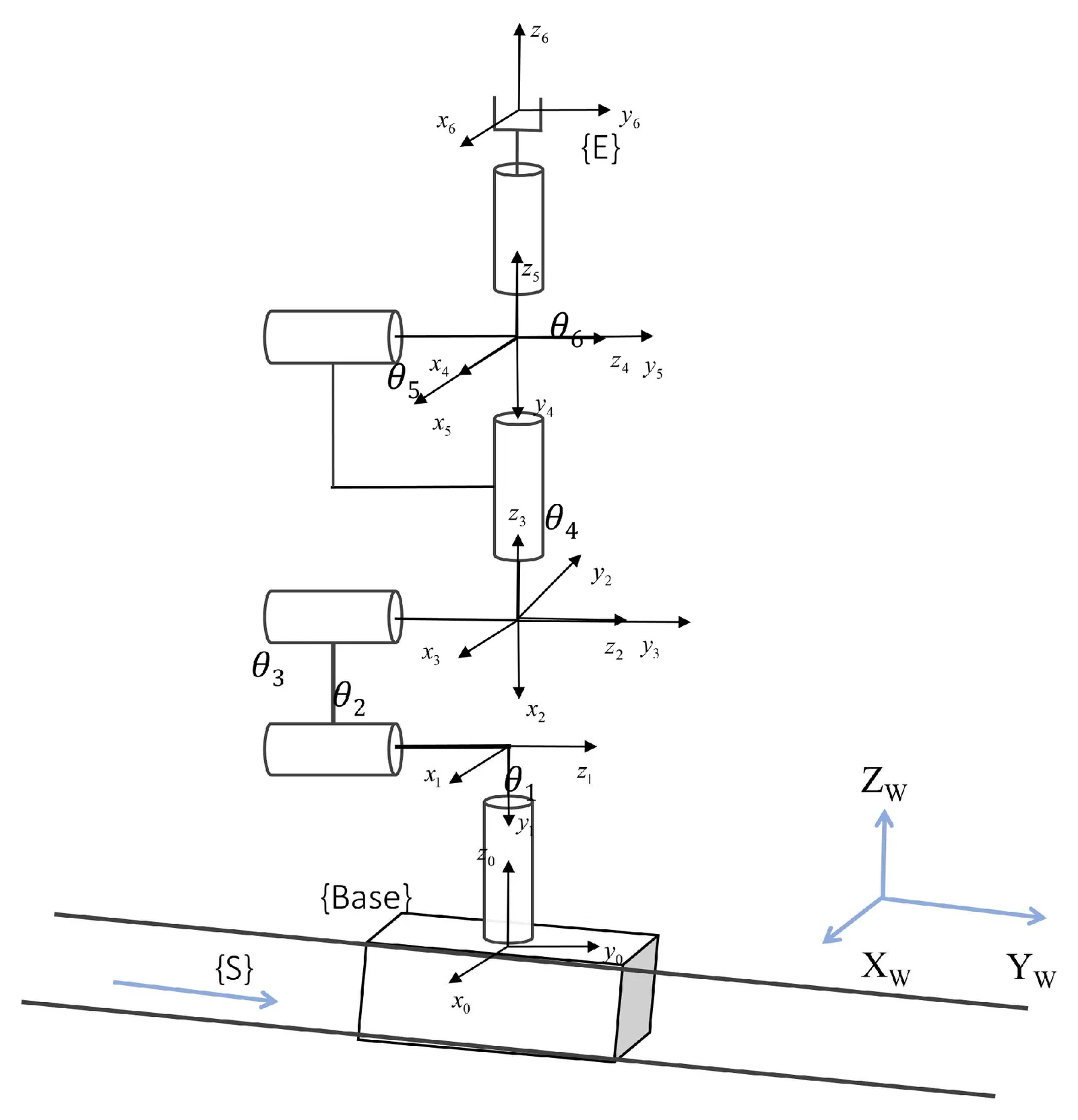
Coordinate systems and DH model of the robotic arm and end-effector.

Here, $q_{0}$ is the rail displacement, and $q_{1},\ldots ,q_{6}$ are the rotational joints of the manipulator. The world frame {W} is fixed at the rail origin, and the manipulator base frame {B} translates with the slider along the rail direction. Figure 3 shows the coordinate frames and the D-H modeling scheme.

The base translation caused by the external rail axis is written as

(3) $$T_{WB}\left(q_{0}\right)=\left[\begin{array}{cccc} 1 & 0 & 0 & q_{0}\\ 0 & 1 & 0 & 0\\ 0 & 0 & 1 & 0\\ 0 & 0 & 0 & 1 \end{array}\right]T_{SB0}$$

where $T_{SB0}$ is the fixed installation transform from the rail coordinate system to the manipulator base when the slider is at its zero position. The standard D-H transform of the ith manipulator joint is [45]

(4) $$A_{i-1,i}=\left[\begin{array}{cccc} cos\theta_{i} & -sin\theta_{i}cos\alpha_{i} & sin\theta_{i}sin\alpha_{i} & a_{i}cos\theta_{i}\\ sin\theta_{i} & cos\theta_{i}cos\alpha_{i} & -cos\theta_{i}sin\alpha_{i} & a_{i}sin\theta_{i}\\ 0 & sin\alpha_{i} & cos\alpha_{i} & d_{i}\\ 0 & 0 & 0 & 1 \end{array}\right]$$

The forward kinematics from the world frame to the end-effector frame {E} is then

(5) $$T_{WE}\left(q\right)=T_{WB}\left(q_{0}\right)\left(\prod_{i=1}^{6}A_{i-1,i}\left(q_{i}\right)\right)T_{6E}$$

The end-effector position vector is

(6) $$p\left(t\right)={\left[x\left(t\right),y\left(t\right),z\left(t\right)\right]}^{T}=trans\left(T_{WE}\left(q\left(t\right)\right)\right)$$

The D-H parameters for the 7-DOF system are listed in Table 1, comprising the external linear rail axis (Joint 0) and the six rotary axes of the manipulator body (Joints 1–6), where *d_i_* denotes link offset, *a_i_* is link length, *α_i_* represents link twist, and *θ_i_* is the joint variable.

### 2.3. Reference Joint-Trajectory Generation and Optimization Problem

Given the prescribed pin sequence and the process requirements of the end-guiding tool, a discrete sequence of reference end-effector poses is first constructed as

(7) $$T=\{T_{1},T_{2},\ldots ,T_{M}\}$$

For each reference pose $T_{k}$, the inverse-kinematics candidate set $S\left(T_{k}\right)$ satisfying joint limits and basic geometric constraints is calculated. A reference configuration is selected using a joint-continuity criterion:

(8) $$q_{k}^{ref}=arg\underset{q\in S\left(T_{k}\right)}{min}{∥q-q_{k-1}^{ref}∥}_{W}^{2}$$

where W is the joint weighting matrix. The discrete reference joint sequence is then assigned initial time intervals and fitted with quintic B-splines to obtain a continuous reference trajectory $q_{ref}\left(t\right)$. The optimization problem is to adjust the execution time and control points of this reference joint trajectory while preserving the pin-passing order and satisfying wiring-process safety constraints, thereby obtaining Pareto-optimal trade-offs among time efficiency, joint smoothness, and harness bending safety. Thus, the optimization problem considered here is the refinement of an executable reference trajectory, not the automatic generation of the wiring route itself.

It should be noted that inverse-kinematic redundancy may influence the obtained joint-space trajectory and the corresponding Pareto-optimal solutions. In this work, multiple IK candidates satisfying joint limits and basic geometric constraints are first calculated for each reference pose, and the reference configuration is then selected using the weighted joint-continuity criterion in Equation (8). This strategy yields a smooth and physically executable reference trajectory while avoiding discontinuous IK-branch switching. The subsequent B-spline optimization searches within a bounded control-point offset range around this selected reference trajectory, as shown in Equation (11). Therefore, the reported Pareto front should be interpreted as the Pareto-optimal solution set conditioned on the selected continuous IK branch. Different feasible IK branches may lead to different joint-motion distributions, and a systematic comparison of IK selection strategies will be investigated in future work.

## 3. Quintic B-Spline Parameterization and Multi-Objective Model

### 3.1. Quintic B-Spline Trajectory Parameterization

The reference joint trajectory is parameterized in normalized time so that the trajectory shape and the total execution duration can be optimized in a unified framework. The relationship between the normalized trajectory parameter and the real execution time, the quintic B-spline [19] representation of the jth joint trajectory, the control-point offset encoding, the decision vector, and the trajectory sampling rule are defined as

(9) t=uT

(10) $$q_{j}\left(u\right)=\sum_{i=1}^{K}P_{j,i}N_{i,5}\left(u\right),\;j=0,1,\ldots ,6$$

(11) $$P_{j,i}=P_{j,i}^{ref}+\Delta P_{j,i}$$

(12) $$x={\left[T,vec\left(\Delta P\right)\right]}^{T},\;D=1+7K$$

(13) $$u_{s}=\frac{s-1}{N_{s}-1},\;t_{s}=u_{s}T,\;s=1,2,\ldots ,N_{s}$$

In Equations (9)–(13), T denotes the total execution time, t is the real execution time, and u is the normalized trajectory parameter. The term $q_{j}(u)$ represents the trajectory of the jth joint, $N_{i,5}(u)$ is the ith quintic B-spline basis function, and $P_{j,i}$ is the corresponding control point. The reference control point and its optimization offset are denoted by $P_{j,i}^{ref}$ and $\Delta P_{j,i}$, respectively, and the decision vector contains T together with all control-point offsets of the rail axis and the six rotational joints. Compared with direct control-point optimization, this offset-based encoding keeps the search region close to the executable reference trajectory while retaining sufficient local freedom for time adjustment, smoothing, and bending-risk reduction. During numerical evaluation, the continuous trajectory is discretized into $N_{s}$ sampling points, and the velocity, acceleration, and jerk of each joint are obtained from the analytical derivatives of the B-spline or approximated by central differences. Since the rail displacement and rotational-joint angles have different units and numerical scales, their derivatives are normalized by the corresponding constraint limits during objective and constraint evaluation.

### 3.2. Objective Functions

The multi-objective model evaluates each candidate trajectory from three aspects: routing efficiency, joint-motion smoothness, and local bending safety. The first two objectives are the total routing time and the normalized joint jerk energy, respectively:

(14) $$f_{1}\left(x\right)=T$$

(15) $$f_{2}\left(x\right)=\frac{1}{N_{s}}\sum_{s=1}^{N_{s}}\sum_{j=0}^{6}{\left(\frac{{\dddot{q}}_{j}\left(t_{s}\right)}{{\dddot{q}}_{j,max}}\right)}^{2}$$

In Equations (14) and (15), $f_{1}$ and $f_{2}$ denote the time objective and the jerk objective, respectively. The jerk term ${\dddot{q}}_{j,\kappa }$ represents the jerk of the jth joint at the sth sampling point, and ${\dddot{q}}_{j,max}$ is the corresponding allowable jerk limit. The normalization by joint-specific limits avoids an unbalanced contribution from the rail axis and rotational joints caused by different physical units. A smaller jerk objective indicates smoother motion and lower servo-tracking shock, which is beneficial for reducing disturbance between the harness and the end-guiding tool.

The third objective is designed to represent local bending risk during pin bypassing. After the end-effector position is obtained from forward kinematics, the sampled trajectory is used to estimate the end-effector velocity, acceleration, local curvature, curvature radius, bending-exceedance penalty, pin-neighborhood weight, and final bending-safety objective as follows:

(16) $$\kappa_{s}=\frac{∥{\dot{p}}_{s}\times {\ddot{p}}_{s}∥}{{∥{\dot{p}}_{s}∥}^{3}+\varepsilon }$$

(17) $$R_{s}=\frac{1}{\kappa_{s}+\varepsilon }$$

(18) $$e_{s}={\left[max\left(0,\frac{R_{min}-R_{s}}{R_{min}}\right)\right]}^{2}$$

(19) $$w_{s}=1+\beta \sum_{k=1}^{N_{p}}exp\left[-\frac{{\left(s-s_{k}\right)}^{2}}{2\sigma_{s}^{2}}\right]$$

(20) $$f_{3}\left(x\right)=\frac{1}{N_{s}}\sum_{s=1}^{N_{s}}w_{s}e_{s}$$

In Equations (16)–(20), $\kappa_{s}$ and $R_{s}$ denote the curvature and curvature radius of the end-effector trajectory at the sth sampling point, respectively. $R_{min}$ is the minimum allowable harness bending radius specified by the wiring process, β denotes the weight amplification coefficient, and $\sigma_{s}$ defines the neighborhood range of each target pin for weighting calculation. The small positive constant ε in the curvature and curvature-radius calculation is introduced to avoid numerical instability at low end-effector velocities. Unlike a globally uniform curvature penalty, the proposed $f_{3}$ emphasizes regions close to pins and sharp turns, where local over-bending and undesired pin contact are more likely to occur.

Actuator torque and energy consumption are not included as independent objectives because the current fixture-board routing task involves light payloads, relatively low speeds, and strict requirements for motion smoothness and local bending safety. The jerk objective partly reflects rapid actuator-load variations, although the optimized trajectories are not claimed to be energy-optimal. Torque- and energy-aware trajectory optimization will be considered in future work for heavier-payload or higher-speed routing tasks.

### 3.3. Constraints and Constraint-Violation Measure

The constraints are divided into robot-motion constraints and wiring-process constraints. The robot-motion constraints include the bounds of the total execution time and control-point offsets, the joint-position limits, and the limits on joint velocity, acceleration, and jerk:

(21) $$T_{min}\leq T\leq T_{max},\;\Delta P_{j,i}^{min}\leq \Delta P_{j,i}\leq \Delta P_{j,i}^{max}$$

(22) $$q_{j}^{min}\leq q_{j}\left(t_{s}\right)\leq q_{j}^{max}$$

(23) $$\left|{\dot{q}}_{j}\left(t_{s}\right)\right|\leq {\dot{q}}_{j,max},\;\left|{\ddot{q}}_{j}\left(t_{s}\right)\right|\leq {\ddot{q}}_{j,max},\;\left|{\dddot{q}}_{j}\left(t_{s}\right)\right|\leq {\dddot{q}}_{j,max}$$

In Equations (21)–(23), $T_{min}$ and $T_{max}$ define the allowable range of the total execution time, while $\Delta P_{j,i}^{min}$ and $\Delta P_{j,i}^{max}$ define the lower and upper bounds of the control-point offsets. The variables $q_{j,s}$, ${\dot{q}}_{j,s}$, ${\ddot{q}}_{j,s}$, and ${\dddot{q}}_{j,s}$ denote the position, velocity, acceleration, and jerk of the jth joint at the sth sampling point. These constraints ensure that the optimized trajectory remains within the physical workspace of the rail–manipulator system and satisfies the safe operating limits of the controller.

The wiring-process constraints are imposed to preserve the prescribed pin-passing sequence and avoid interference with the fixture board and non-target pins. The pin-passing constraint, board-clearance constraint, and non-target-pin safety-distance constraint are expressed as

(24) $$\underset{s\in \Omega_{k}}{min}{∥p\left(t_{s}\right)-P_{k}∥}_{2}\leq \delta_{p},\;k=1,2,\ldots ,N_{p}$$

(25) $$p_{z}\left(t_{s}\right)-z_{b}\geq h_{clr}$$

(26) $${∥p_{xy}\left(t_{s}\right)-P_{i,xy}∥}_{2}\geq r_{pin}+r_{tool}+r_{safe},\;i\in O\left(s\right)$$

In Equations (24)–(26), $\Omega_{k}$ denotes the sampling window associated with the kth target pin, $P_{k}$ is the prescribed passing or bypassing reference point, and $\delta_{p}$ is the allowable pin-passing error. The parameters $z_{b}$ and $h_{clr}$ represent the fixture-board height and the required minimum clearance, respectively. The set $O\left(s\right)$ contains the non-target pins considered at the sth sampling point, and $r_{safe}$ is the required minimum distance between the end-guiding tool and these pins. Together, these constraints ensure that the optimized trajectory follows the specified routing order, maintains board clearance, and avoids unintended contact with surrounding pins.

For unified constraint handling in the evolutionary optimization process, all constraints are converted into the standard inequality form $g_{r}(x)\leq 0$. The violation of each constraint and the total constraint violation of a candidate solution are calculated as

(27) $$v_{m}\left(x\right)=max\{0,g_{m}\left(x\right)\}$$

(28) $$\phi \left(x\right)=\sum_{m=1}^{M_{c}}\omega_{m}v_{m}^{2}\left(x\right)$$

In Equations (27) and (28), $v_{m}\left(x\right)$ is the non-negative violation value of the mth constraint, and $\omega_{m}$ is its corresponding weight. Each violation term is normalized by its physical limit or tolerance before aggregation; therefore, the weights reflect the relative priority of different constraint groups rather than differences in units. Higher weights are assigned to wiring-process constraints because violations of pin passing, board clearance, or non-target-pin distance directly invalidate the routing task. The total constraint violation is used only to compare infeasible individuals under the feasibility-priority rule, whereas feasible individuals are ranked solely according to the three objective functions. This treatment limits the influence of the selected weights on the final feasible Pareto set.

## 4. Constraint-Guided Adaptive DE-NSGA-II Algorithm

### 4.1. Overall Framework

Standard NSGA-II has strong multi-objective search ability, but it is easily affected by the narrow feasible region and strongly coupled constraints in the proposed wiring problem. Therefore, this paper proposes a constraint-guided adaptive DE-NSGA-II algorithm that combines the continuous-variable search ability of differential evolution, the elitist non-dominated sorting mechanism of NSGA-II, and a repair strategy designed for wiring-process constraints. The main improvements are as follows:

1. control-point offset encoding is used so that the population searches around the executable reference trajectory and avoids invalid individuals far from the feasible route;
2. the DE mutation strategy is adaptively switched according to the feasible rate of the current generation, and the scaling factor $F_{i}$ and crossover probability $CR_{i}$ are dynamically updated;
3. a hierarchical repair operator is designed, including boundary projection, time scaling, local smoothing, pin-neighborhood risk repair, and passing-error repair;
4. a feasibility-priority rule and NSGA-II elitist preservation are used to balance constraint satisfaction, convergence, and diversity.

### 4.2. Adaptive DE Mutation and Crossover

In generation g, the population is denoted by $\{x_{i}^{g}{\}}_{i=1}^{N_{pop}}$. The feasible rate of the current generation is first calculated as

(29) $$r_{f}^{g}=\frac{\left|\left\{x_{i}^{g}:\phi \left(x_{i}^{g}\right)=0\right\}\right|}{N_{pop}}$$

When $r_{f}^{g}$ is low, the population has not sufficiently entered the feasible region. The algorithm mainly uses the DE/rand/1 strategy to enhance exploration:

(30) $$v_{i}^{g}=x_{r1}^{g}+F_{i}^{g}\left(x_{r2}^{g}-x_{r3}^{g}\right)$$

When $r_{f}^{g}$ is high, the feasible region has been reasonably covered. The proportion of the DE/current-to-best/1 strategy is increased to enhance exploitation near high-quality non-dominated solutions:

(31) $$v_{i}^{g}=x_{i}^{g}+F_{i}^{g}\left(x_{best}^{g}-x_{i}^{g}\right)+F_{i}^{g}\left(x_{r1}^{g}-x_{r2}^{g}\right)$$

where r1, r2, and r3 are mutually different random indices, and $x_{best}^{g}$ can be selected from the current feasible non-dominated set. Binomial crossover is performed for each dimension:

(32) $$u_{i,d}^{g}=\left\{\begin{array}{ll} v_{i,d}^{g}, & rand\left(0,1\right)\leq CR_{i}^{g}\;or\;d=d_{rand},\\ x_{i,d}^{g}, & otherwise. \end{array}\right.$$

where $d_{rand}$ ensures that at least one dimension comes from the mutation vector. Parameters $F_{i}$ and $CR_{i}$ are adaptively updated with probabilities $\tau_{F}$ and $\tau_{CR}$, and their ranges are adjusted for different search stages to balance exploration and exploitation.

### 4.3. Hierarchical Repair and Feasibility-Priority Selection

To avoid directly discarding a large number of infeasible individuals, hierarchical repair is performed after trial individuals are generated. First, T and $\Delta P_{j,i}$ are projected into their box bounds. Second, when velocity, acceleration, or jerk exceeds its limit, time scaling is applied while keeping the geometric shape of the trajectory approximately unchanged. Let the three dynamic exceedance factors be $\eta_{v}$, $\eta_{a}$, and $\eta_{j}$. The time-scaling factor is defined as

(33) $$\gamma =max\left(1,\eta_{v},\sqrt{\eta_{a}},\sqrt[{3}]{\eta_{j}}\right),\;T\leftarrow \gamma T$$

Next, for local jerk peaks or curvature changes, adjacent control points are smoothed:

(34) $$P_{j,i}\leftarrow \left(1-2\lambda \right)P_{j,i}+\lambda P_{j,i-1}+\lambda P_{j,i+1},\;0<\lambda <0.5$$

For bending risk in pin neighborhoods, the risk score is defined as

(35) $$\rho_{s}=w_{s}max\left(0,R_{min}-R_{s}\right)$$

Local control point intervals with large $\rho_{s}$ values are preferentially adjusted or smoothed. If the pin-passing error remains out of tolerance after repair, local control points in the corresponding window are slightly corrected, after which the objective functions and total constraint violation are recalculated.

One-to-one selection between the parent individual $X_{i}^{g}$ and trial individual $u_{i}^{g}$ follows a feasibility-priority rule: a feasible solution is preferred to an infeasible solution; if both are infeasible, the individual with a smaller ∅ is selected; if both are feasible, Pareto dominance is used, and mutually non-dominated individuals are placed in the merged pool. Fast non-dominated sorting and crowding-distance calculation are then performed on the merged pool; the next-generation population is truncated from it, and the external elite archive is updated.

### 4.4. Algorithm Workflow

**Figure 4:**
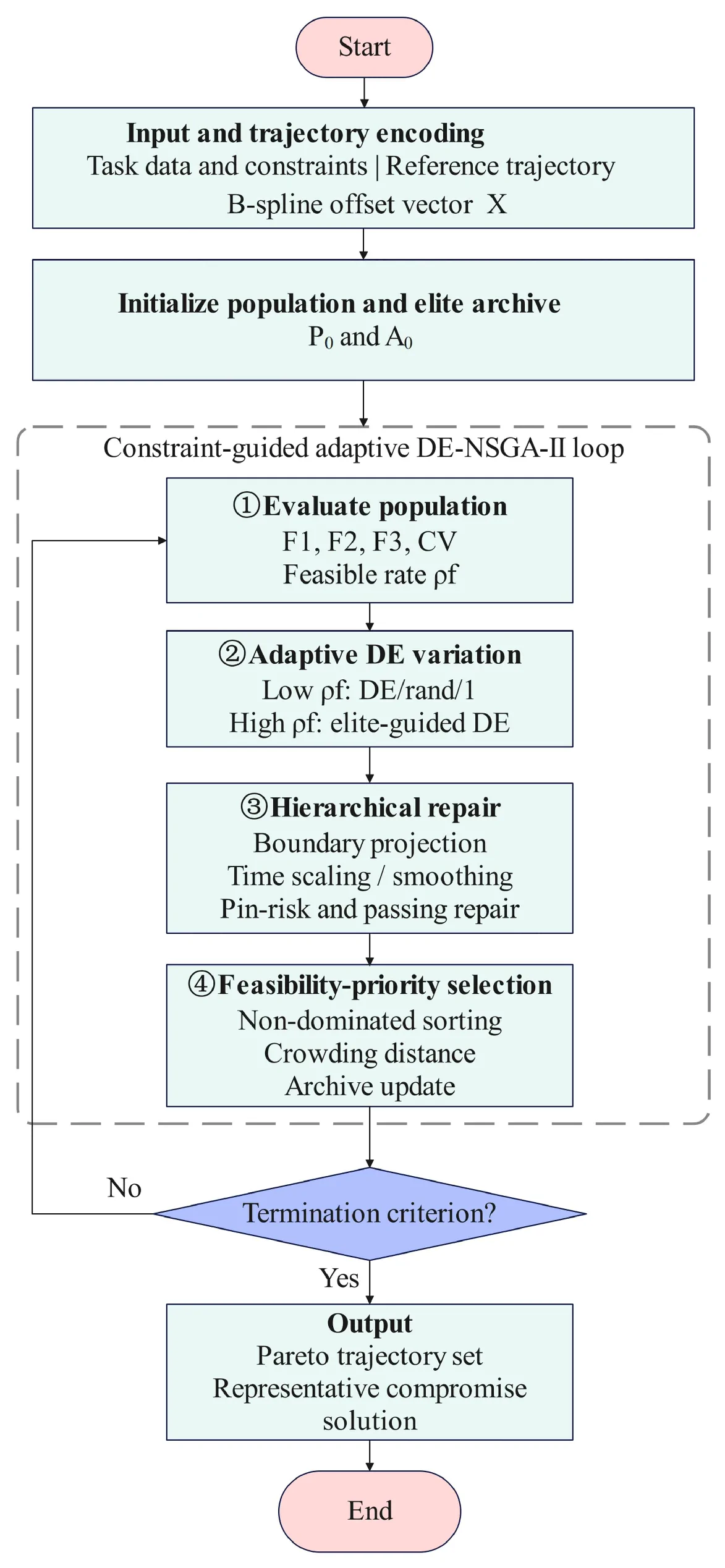
Compact workflow of the proposed constraint-guided adaptive DE-NSGA-II algorithm.

The complete workflow of the constraint-guided adaptive DE-NSGA-II algorithm is shown in Figure 4. Compared with standard NSGA-II, the proposed workflow integrates feasible-rate-driven DE search, hierarchical repair, and feasibility-priority elitist preservation into a closed loop. The algorithm can adjust its search behavior according to the exploration state of the feasible region and allocate more optimization resources to high-quality feasible trajectories.

## 5. Simulation and Experimental Validation

### 5.1. Numerical Simulation Settings and Comparative Algorithms

A seven-axis wiring-robot simulation environment was built in MATLAB R2024a. It includes forward and inverse kinematics, quintic B-spline trajectory generation, objective-function evaluation, constraint checking, and multi-objective optimization. A typical routing path on an aircraft harness fixture board was selected as the test task. It contains six key path points: the starting point, four intermediate points near pins, and the terminal point. The effective board length is 5000 mm, and the routing-region width is 800 mm.

To ensure reproducibility, the task parameters, constraint thresholds, and algorithm parameters were set uniformly, as listed in Table 2. The total execution time was searched within 12–24 s, and the initial reference time was 20 s. The quintic B-spline degree was 5, the number of control points was 12, and the number of trajectory-evaluation sampling points was 1000. The value of 12 control points was selected as a compromise between trajectory representation capability and optimization cost. Since the spline degree is 5, sufficient control points are required to describe local bypassing motions around pins and to adjust curvature in pin-neighborhood regions. For the six-key-point routing task, 12 control points provide adequate local adjustment freedom to preserve the prescribed pin-passing order while allowing trajectory smoothing and bending-risk reduction. In preliminary parameter tuning, fewer control points reduced the local adjustment capability and made it more difficult to satisfy pin-passing and bending-risk constraints, whereas more control points increased the decision-space dimension and computational cost without obvious improvement in the optimized trajectory. Therefore, $N_{c}=12$ was used for the baseline routing case. For more complex layouts with more key path points or routing branches, $N_{c}$ can be increased according to routing complexity to provide additional trajectory flexibility. The control-point offset bounds of the rail axis and the six rotational joints were set according to the values listed in Table 2. The joint-position limits were consistent with Table 1, and the velocity, acceleration, and jerk limits were set according to the safe operating range of the controller.

**Figure 5:**
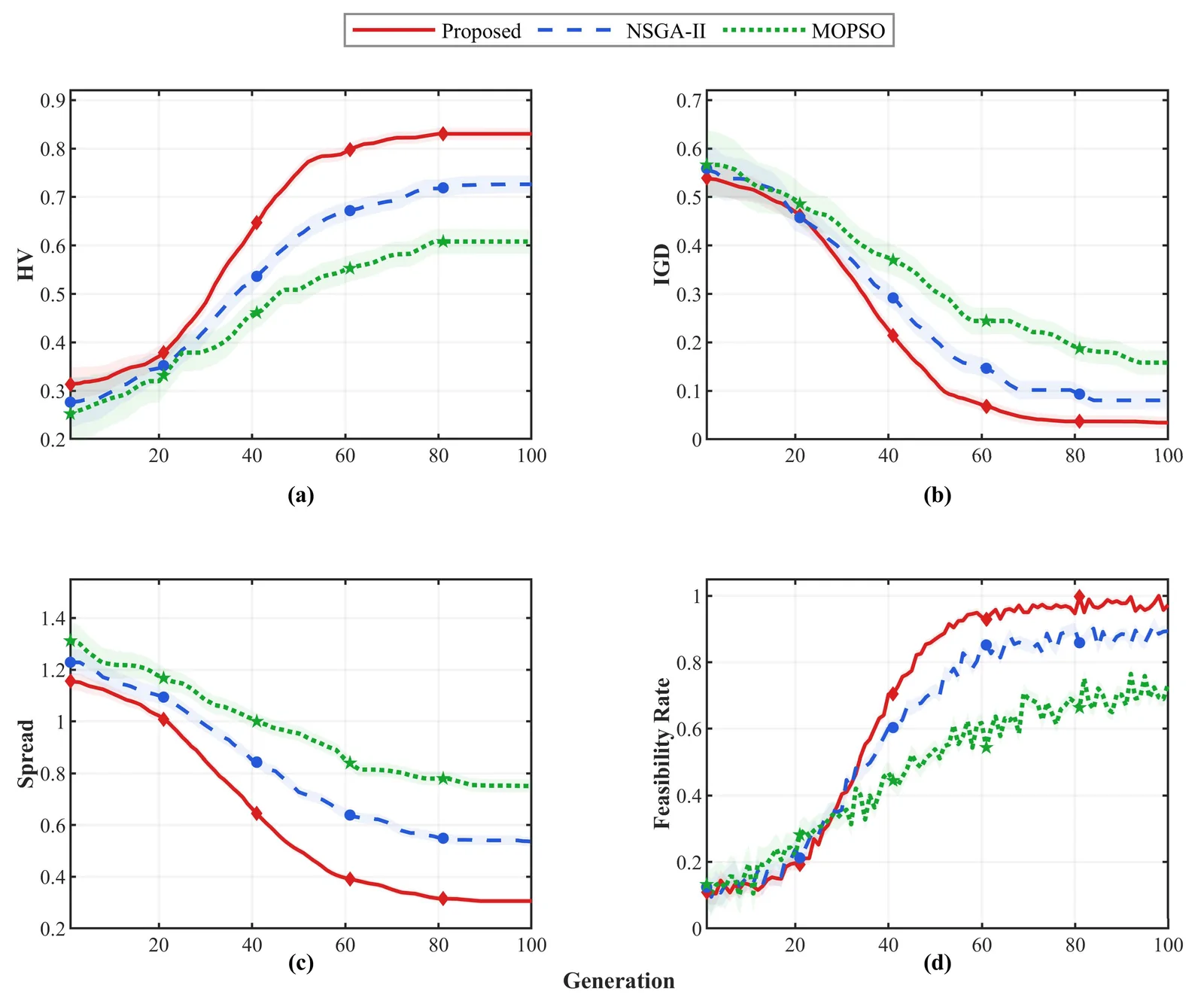
Multi-objective optimization performance of different algorithms: (**a**) HV; (**b**) IGD; (**c**) Spread; (**d**) feasibility rate.

The comparative algorithms were standard NSGA-II, MOPSO, and the proposed constraint-guided adaptive DE-NSGA-II. The three algorithms used the same initial reference trajectory, constraints, and maximum number of function evaluations. The maximum number of evaluations in a single run was approximately $4.0\times 10^{4}$. Hypervolume (HV), inverted generational distance (IGD), Spread, and feasibility rate were used to evaluate algorithm performance, as shown in Figure 5.

As shown in Figure 5, the proposed algorithm converges to an HV of 0.84 and reduces IGD to 0.04, outperforming NSGA-II (0.72/0.09) and MOPSO (0.61/0.16). This indicates better front approximation and coverage in the three-objective space. The Spread value of the proposed algorithm converges to 0.30, indicating a more uniform solution distribution. By generation 80, its feasible rate reaches 97%, higher than that of NSGA-II (88%) and MOPSO (72%). These results show that the proposed constraint handling and adaptive search mechanisms improve both the feasible-solution ratio and Pareto-set quality in the strongly constrained trajectory optimization problem.

### 5.2. Ablation Study of the Proposed Algorithm

To further quantify the contribution of each mechanism in the proposed constraint-guided adaptive DE-NSGA-II algorithm, an ablation study was conducted under the same routing task, constraints, population size, maximum number of generations, and independent-run settings as those used in Section 5.1. Four simplified variants were constructed by removing one component at a time from the full algorithm: (1) *w*/*o* CPO, in which the control-point offset encoding was replaced by direct control-point encoding; (2) *w*/*o* FRD-DE, in which the feasible-rate-driven adaptive DE mutation was replaced by a fixed DE/rand/1 strategy with constant parameters; (3) *w*/*o* HR, in which the hierarchical repair operator was removed except for basic boundary projection; and (4) *w*/*o* FPS, in which the feasibility-priority selection rule was replaced by ordinary objective-based non-dominated sorting with penalty-based constraint handling. The hypervolume (HV), inverted generational distance (IGD), Spread, and feasibility rate were used as evaluation metrics.

The ablation results are summarized in Table 3. The full algorithm achieves the best overall performance in terms of HV, IGD, Spread, and feasibility rate. Removing the control-point offset encoding reduces the early feasible-solution ratio because the population is no longer initialized and searched around an executable reference trajectory. Removing the feasible-rate-driven DE strategy mainly degrades convergence quality, as indicated by lower HV and higher IGD, which shows that the adaptive transition between exploration and exploitation is beneficial for the constrained trajectory optimization problem. Removing the hierarchical repair operator causes the most significant decrease in feasibility rate, confirming its role in recovering individuals that violate dynamic, pin-passing, clearance, and bending-risk constraints. Removing the feasibility-priority selection rule also decreases the final feasibility rate and Pareto-front quality, indicating that constraint satisfaction must be explicitly considered during elitist preservation.

These results demonstrate that the proposed performance improvement is not caused by a single mechanism alone. Instead, the feasible-rate-driven DE search, hierarchical repair, feasibility-priority selection, and control-point offset encoding play complementary roles in improving convergence, diversity, and feasible-solution search in the strongly constrained wiring-trajectory optimization problem.

### 5.3. Quintic B-Spline Trajectory and Joint Motion Characteristics

To analyze the effect of quintic B-spline trajectory parameterization on joint-motion smoothness, a set of end-effector key path points was generated according to the wiring process, and the corresponding joint-space path points were obtained through inverse kinematics, as shown in Table 4. The seventh axis represents the linear-rail displacement in millimeters, while the remaining six variables are manipulator joint angles in degrees. In the table, $P_{0}$ is the initial pose, $P_{5}$ is the target pose, and $P_{1}$–$P_{4}$ are intermediate path points.

**Figure 6:**
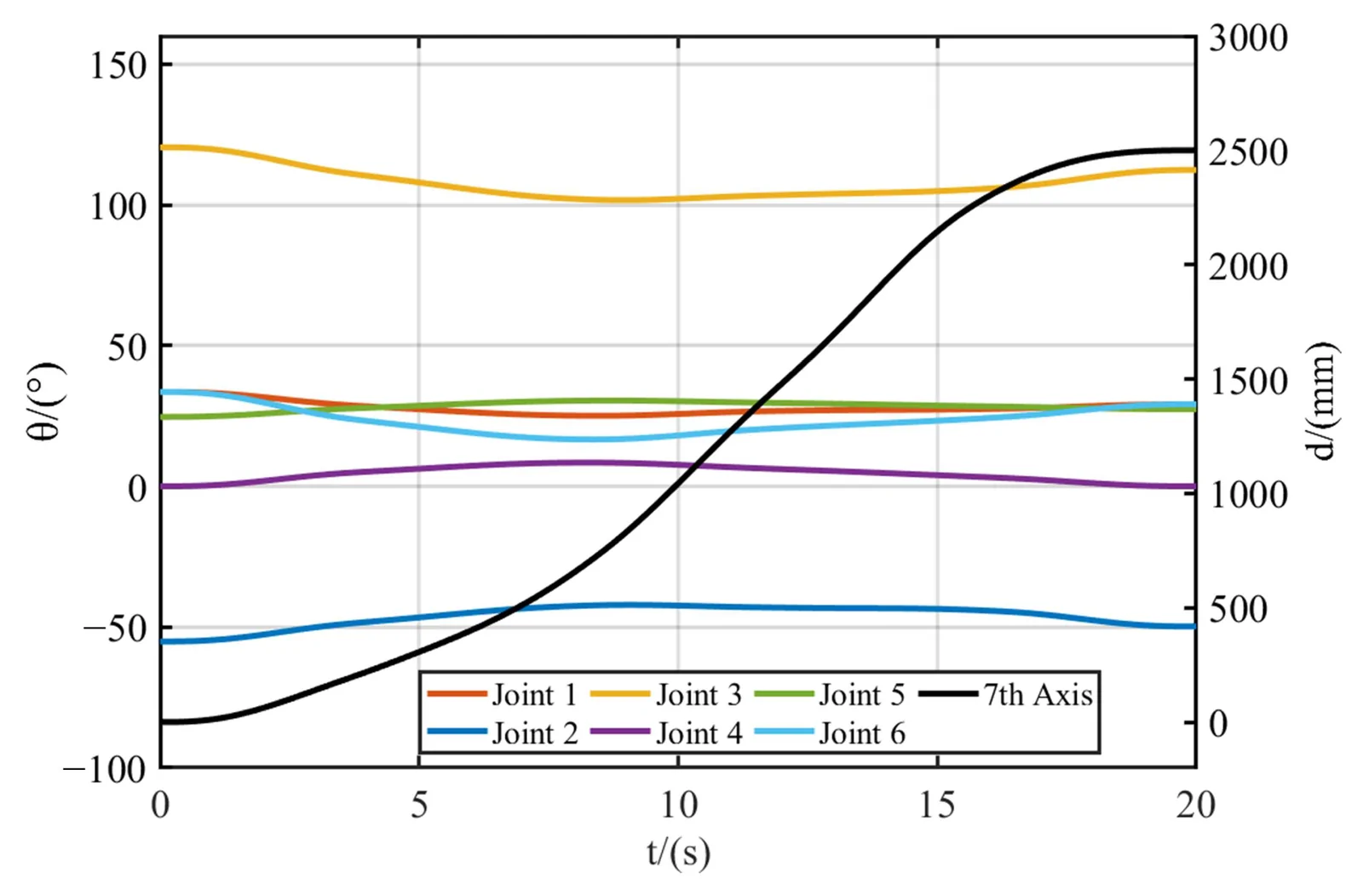
Joint position curves generated by quintic B-spline trajectory planning.

**Figure 7:**
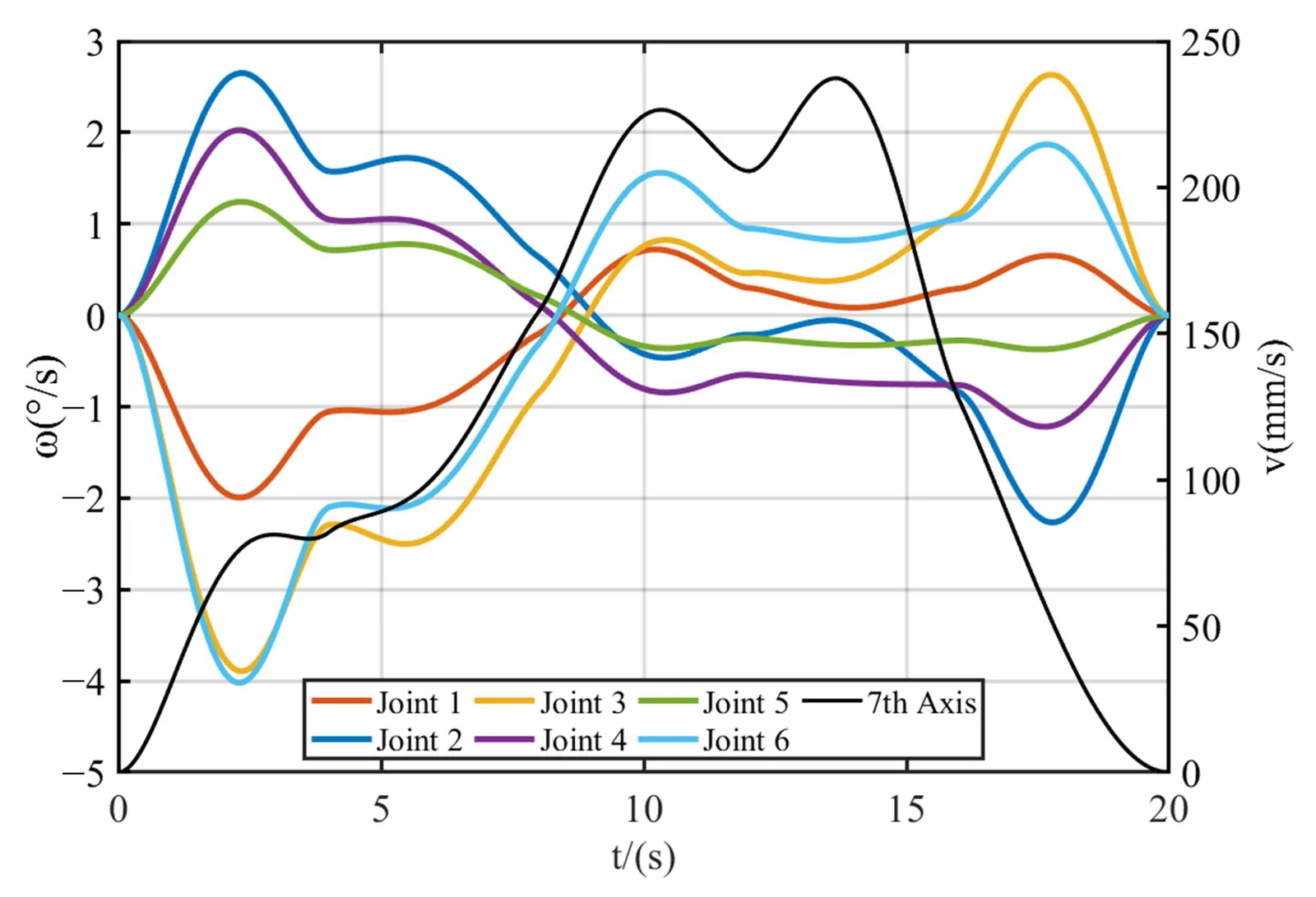
Joint velocity curves generated by quintic B-spline trajectory planning.

**Figure 8:**
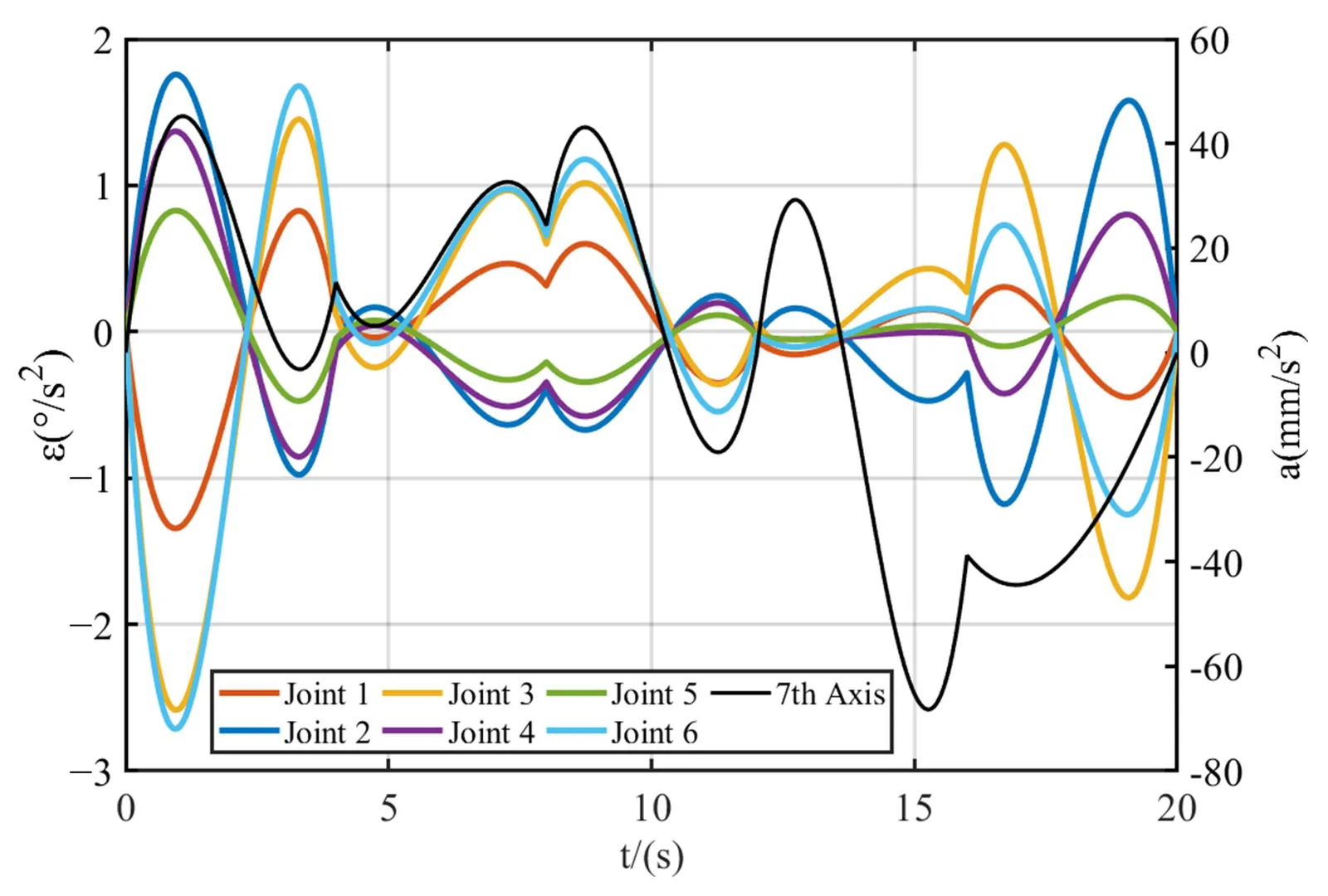
Joint acceleration curves generated by quintic B-spline trajectory planning.

In the initial trajectory generation, the time interval between neighboring key path points was set to 4 s, resulting in a total reference time of 20 s. The velocity and acceleration of each joint at t=0 s and t=20 s were constrained to zero to reduce start–stop shock. Based on these conditions, quintic B-spline trajectories were solved in MATLAB. The resulting joint position, velocity, and acceleration curves are shown in Figure 6, Figure 7 and Figure 8.

Figure 6, Figure 7 and Figure 8 show that the joint position curves smoothly connect neighboring path points, while the velocity and acceleration curves vary continuously and satisfy the zero-velocity and zero-acceleration constraints at the initial and final times. The seventh axis mainly covers the large fixture-board workspace, whereas the six rotational joints adjust the pose and local bypassing position of the end-guiding tool near pins. The quintic B-spline trajectory avoids velocity and acceleration discontinuities caused by directly connecting discrete path points, providing a smooth, continuous, and constraint-checkable trajectory representation for multi-objective optimization.

The subsequent optimization does not directly change the discrete path points. Instead, it searches near the reference B-spline control points using offset encoding. This preserves the executability and routing order of the initial inverse-kinematics trajectory while reducing invalid search regions, improving the early feasible-solution ratio, and suppressing local oscillations.

### 5.4. Pareto Front and Convergence Analysis

**Figure 9:**
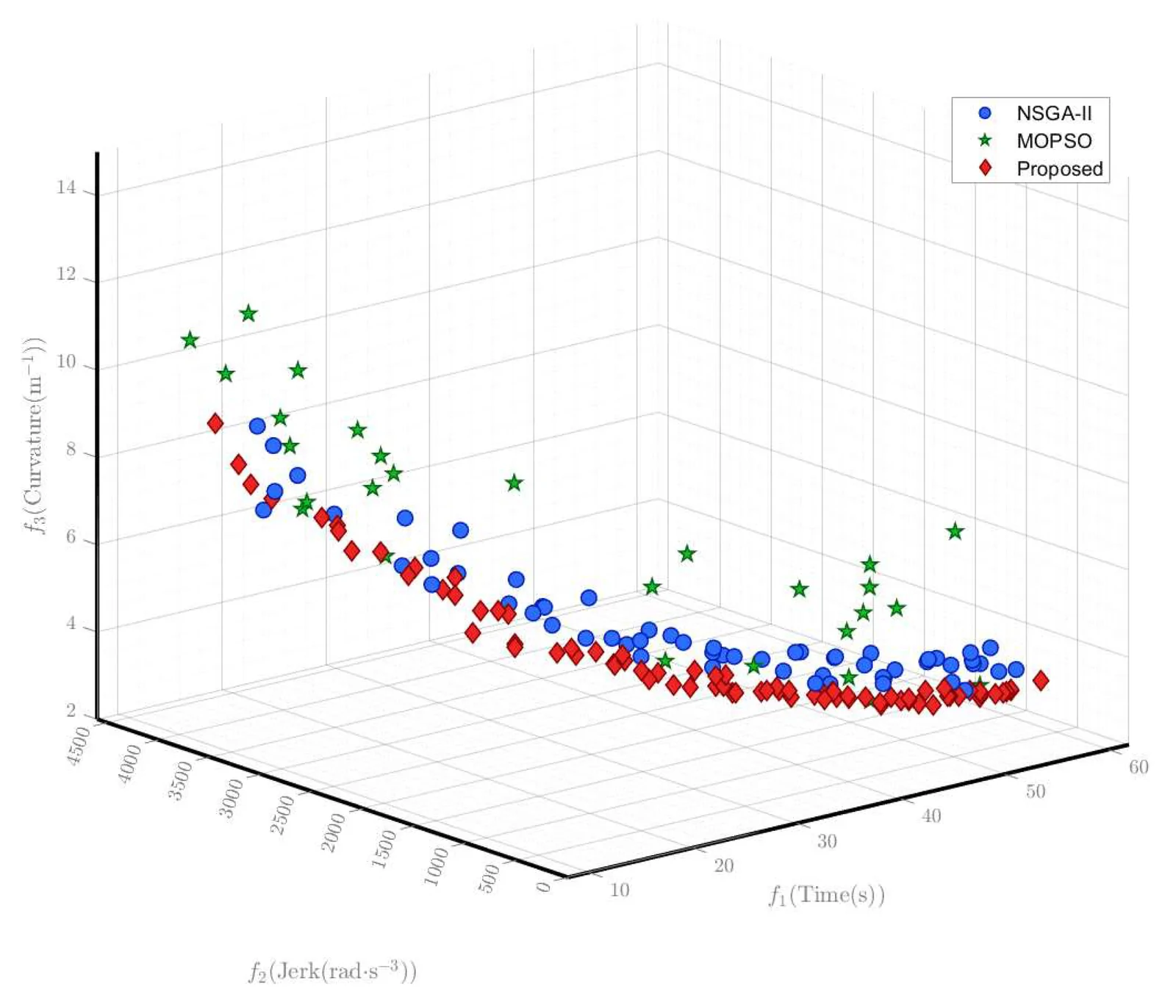
Pareto fronts and representative solutions obtained by different algorithms.

Figure 9 shows the Pareto solution sets obtained by different algorithms for the typical routing task. Overall, the proposed algorithm forms a more continuous trade-off distribution among execution time, normalized jerk energy, and the pin-neighborhood bending-safety objective. For the same execution-time level, it provides lower normalized jerk energy and a smaller bending-safety proxy. For a similar smoothness level, it also offers shorter execution-time options.

These results are mainly due to three factors. First, control-point offset encoding makes the initial search region close to the executable reference trajectory. Second, the adaptive DE strategy improves both global exploration and local exploitation in the continuous decision-variable space. Third, the hierarchical repair operator reduces the consumption of search resources by infeasible individuals. In comparison, standard NSGA-II increases the feasible-solution ratio more slowly in the strongly constrained scenario, and MOPSO is more sensitive to parameter settings and tends to cluster particles in local regions. Therefore, the proposed algorithm provides better overall performance in Pareto-front quality, convergence stability, and solution diversity.

Representative Pareto solutions further show that the proposed algorithm suppresses jerk peaks in pin-bypassing and sharp-turning regions while maintaining the prescribed pin-passing, clearance, and non-target-pin-distance constraints. Therefore, the obtained Pareto solutions are not only better distributed in the objective space but also more suitable for physical execution under wiring-process constraints.

### 5.5. Digital-Twin Pre-Validation and Physical Platform Experiment

**Figure 10:**
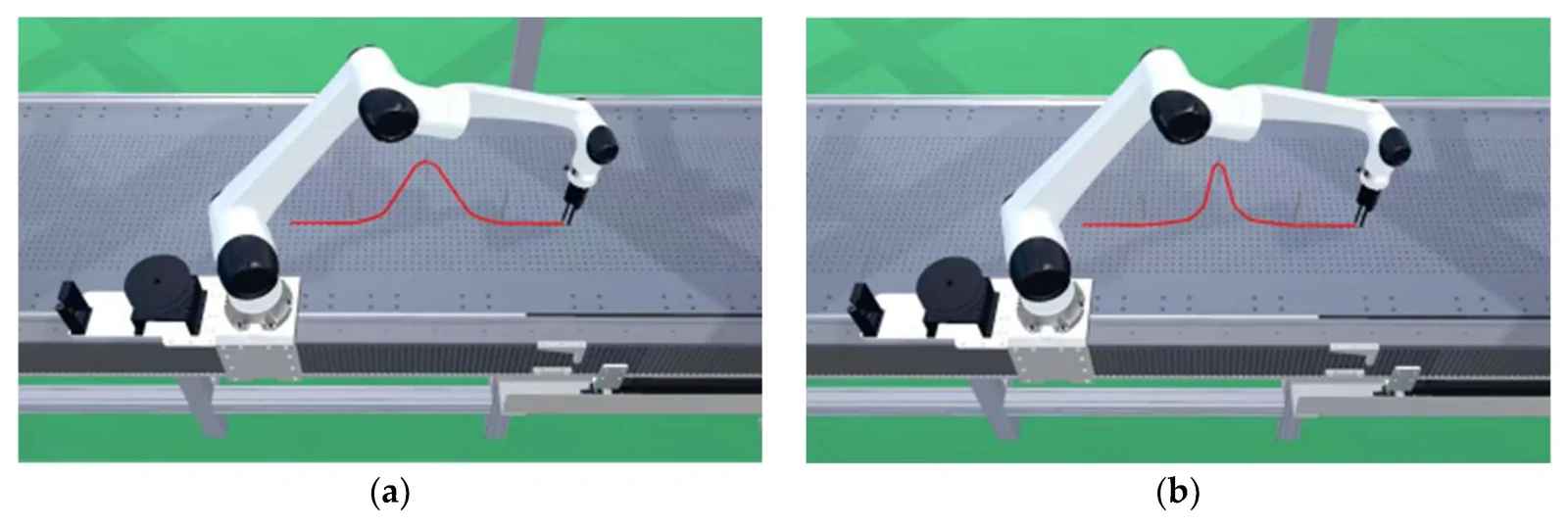
Three-dimensional visualization of different trajectories in the Unity3D digital-twin system: (**a**) optimized trajectory considering pin-neighborhood bending safety; (**b**) comparison trajectory without the bending-safety objective.

Digital-twin validation is used as a risk pre-check between offline simulation and physical execution. A Unity3D 2022.1.15f1 virtual scene was constructed, including the linear rail, six-DOF manipulator, fixture board, pin array, end-guiding tool, and harness model. The joint trajectory output by MATLAB was converted into time-series data to drive the virtual robot. Digital-twin replay was used to check whether the end effector bypassed pins in the specified order, whether board clearance was satisfied, whether potential collisions occurred near non-target pins, and whether the curvature changes in key turning regions were reasonable. Figure 10 shows the digital-twin visualization results for representative Pareto solutions.

As shown in Figure 10, after the pin-neighborhood bending-safety objective is introduced, the end-effector trajectory becomes smoother around pins, reducing the risk of local over-bending. Without this objective, the trajectory exhibits higher curvature in some pin neighborhoods, which may lead to excessive cable bending or undesired pin contact.

**Figure 11:**
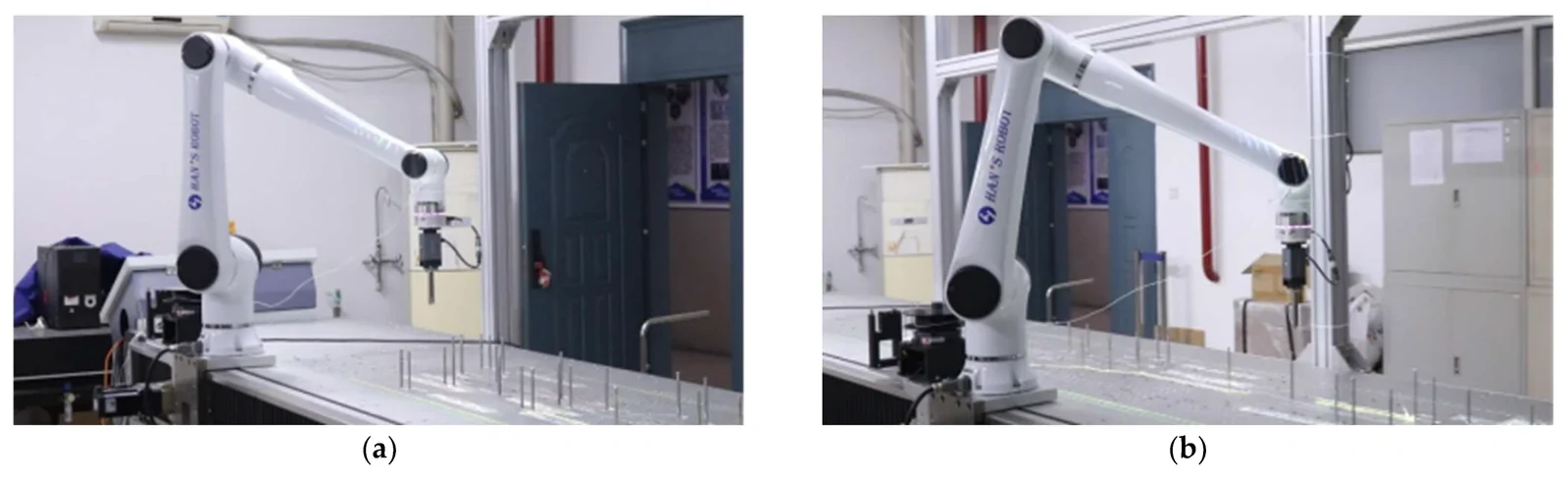

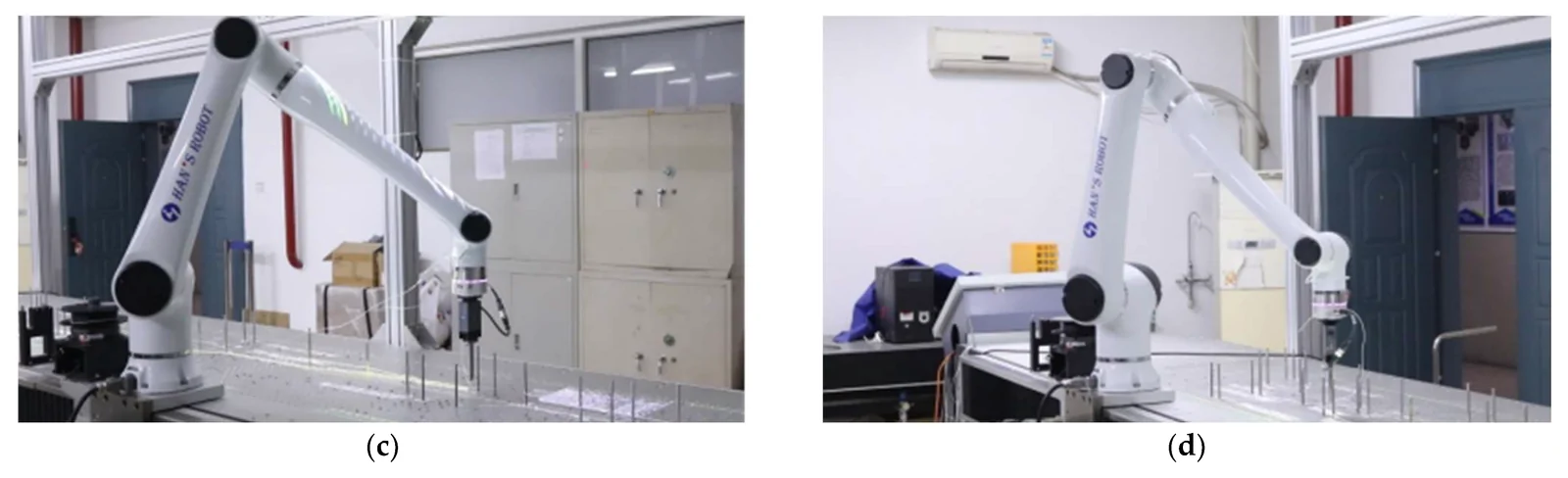
Representative physical validation sequence for the six-key-point benchmark case: (**a**) initial pose; (**b**) transition pose 1; (**c**) transition pose 2; (**d**) target pose. Additional validation cases covering up to 18 key points and four branches are summarized in Table 6.

In the physical platform experiment, representative feasible solutions obtained by MOPSO, standard NSGA-II, and the proposed algorithm were sent to the seven-axis wiring robot platform for execution, as shown in Figure 11. The recorded data included execution time, joint commands and servo feedback, maximum velocity/acceleration/jerk, minimum distance between the end effector and pins, final harness-forming quality, and controller alarm state. All three methods were able to complete the basic routing task in successful trials, but the proposed method showed better motion smoothness, safety distance, and repeated-execution stability.

To further quantify the digital-twin pre-validation and physical platform results, representative feasible solutions from the three methods were each executed ten times. The physical execution time, normalized jerk energy, bending-safety proxy, maximum passing error, minimum board clearance, minimum distance to non-target pins, repeated-execution success rate, and number of controller alarms were recorded. The statistics are listed in Table 5.

**Table 5:** Repeated physical-platform experimental results for the three methods.

Method	Time (s)	Normalized Jerk	Bending Index	Passing Error (mm)	Board Clearance (mm)	Pin Distance (mm)	Success Rate
MOPSO	14.91 ± 0.08	0.338 ± 0.026	0.061 ± 0.012	2.81 ± 0.23	31.6 ± 1.4	41.3 ± 2.7	8/10
NSGA-II	16.34 ± 0.06	0.294 ± 0.018	0.038 ± 0.009	2.35 ± 0.18	34.8 ± 1.2	45.8 ± 2.1	9/10
Proposed	15.72 ± 0.05	0.217 ± 0.014	0.014 ± 0.004	1.62 ± 0.11	38.7 ± 1.0	53.5 ± 1.8	10/10

Table 5 shows that MOPSO obtains the shortest execution time but has higher normalized jerk energy and a higher bending-safety proxy, indicating remaining local motion shock and over-bending risk near pins. Standard NSGA-II improves smoothness and safety margins relative to MOPSO but still produces one alarm or temporary adjustment in repeated execution. In contrast, the proposed method is not the fastest, but it achieves better jerk energy, bending safety, passing accuracy, and safety distance. No controller alarm occurs in ten repeated trials, confirming a more stable overall balance among time efficiency, trajectory smoothness, and wiring-process safety.

To substantiate the observed performance differences, pairwise statistical significance tests were conducted on the ten repeated experimental results for each metric. Specifically, two-sample *t*-tests were applied to the execution time, normalized jerk, and bending index between the proposed method and each comparison algorithm. The results indicate that the improvements achieved by the proposed method over MOPSO and NSGA-II are statistically significant for normalized jerk (*p* < 0.01 for both comparisons) and bending index (*p* < 0.01 for both comparisons). For execution time, the difference between the proposed method and NSGA-II is also significant (*p* < 0.05), while the comparison with MOPSO yields *p* ≈ 0.08, suggesting a marginal but not fully significant advantage of MOPSO in raw speed under the current sample size.

This apparent trade-off, where MOPSO achieves a marginally faster execution but with significantly higher jerk energy (0.338 vs. 0.217) and a much larger bending-safety proxy (0.061 vs. 0.014), is critical for the target application. In aircraft harness routing, the primary engineering objective is not simply minimizing cycle time but ensuring consistent process quality and reliability. High jerk excitations can lead to servo tracking errors and cause abrupt tension variations in the harness, while a large bending index directly correlates with the risk of local over-bending and insulation damage, which are unacceptable quality defects. The proposed method’s marginally longer execution time (approximately 5.4% slower than MOPSO) is therefore a justifiable investment, as it yields a statistically and practically significant improvement in trajectory smoothness (35.8% reduction in normalized jerk) and bending safety (77.0% reduction in the bending-safety proxy), while also guaranteeing 100% successful, alarm-free execution. This balanced performance, prioritizing process safety over raw speed, is more aligned with the reliability-driven requirements of aircraft wire-harness manufacturing.

In addition to the six-point routing case detailed above, the proposed method was further validated on four additional fixture-board layouts with varying complexity, as summarized in Table 6. These experiments were conducted on the same 5000 mm × 800 mm fixture board under identical platform conditions and controller settings, covering a broader range of harness routing scenarios encountered in actual production. For each test case, the proposed algorithm was executed with consistent parameter configurations, and the resulting trajectories were physically executed on the robot platform.

As shown in Table 6, the proposed framework successfully generated executable trajectories for all test cases, with success rates ranging from 90% to 100% over ten repeated runs and no controller alarms reported. Notably, Case 5 contains 18 key points and four branches, representing a significantly higher complexity than the six-point case presented in Table 4. The consistent performance across these diverse configurations—varying in the number of via points, branch topology, and pin spatial distribution—supports the generalizability of the proposed method to more complex harness layouts. While the detailed quantitative metrics (jerk energy and bending index, etc.) are reported for the six-point case as a representative benchmark, the additional cases confirm that the algorithm reliably converges to feasible solutions without requiring case-specific parameter tuning. The board clearance, pin-passing accuracy, and non-target pin avoidance constraints were satisfied in all successful runs.

### 5.6. Vision-Based Validation of the Bending-Safety Proxy

To further verify whether the proposed bending-safety proxy $f_{3}$ can reflect the actual bending state of the harness, a vision-based validation experiment was conducted on the physical platform. During execution, an industrial camera was used to capture the local harness shape near the outlet of the guiding tool and in pin-neighborhood regions. The harness centerline was extracted from the images, and the actual bending radius $R_{harn}$ was obtained by local circular-arc fitting. Meanwhile, the robot joint data were synchronously recorded, and the corresponding end-effector curvature radius $R_{end}$ was calculated through forward kinematics and trajectory differentiation.

**Figure 12:**
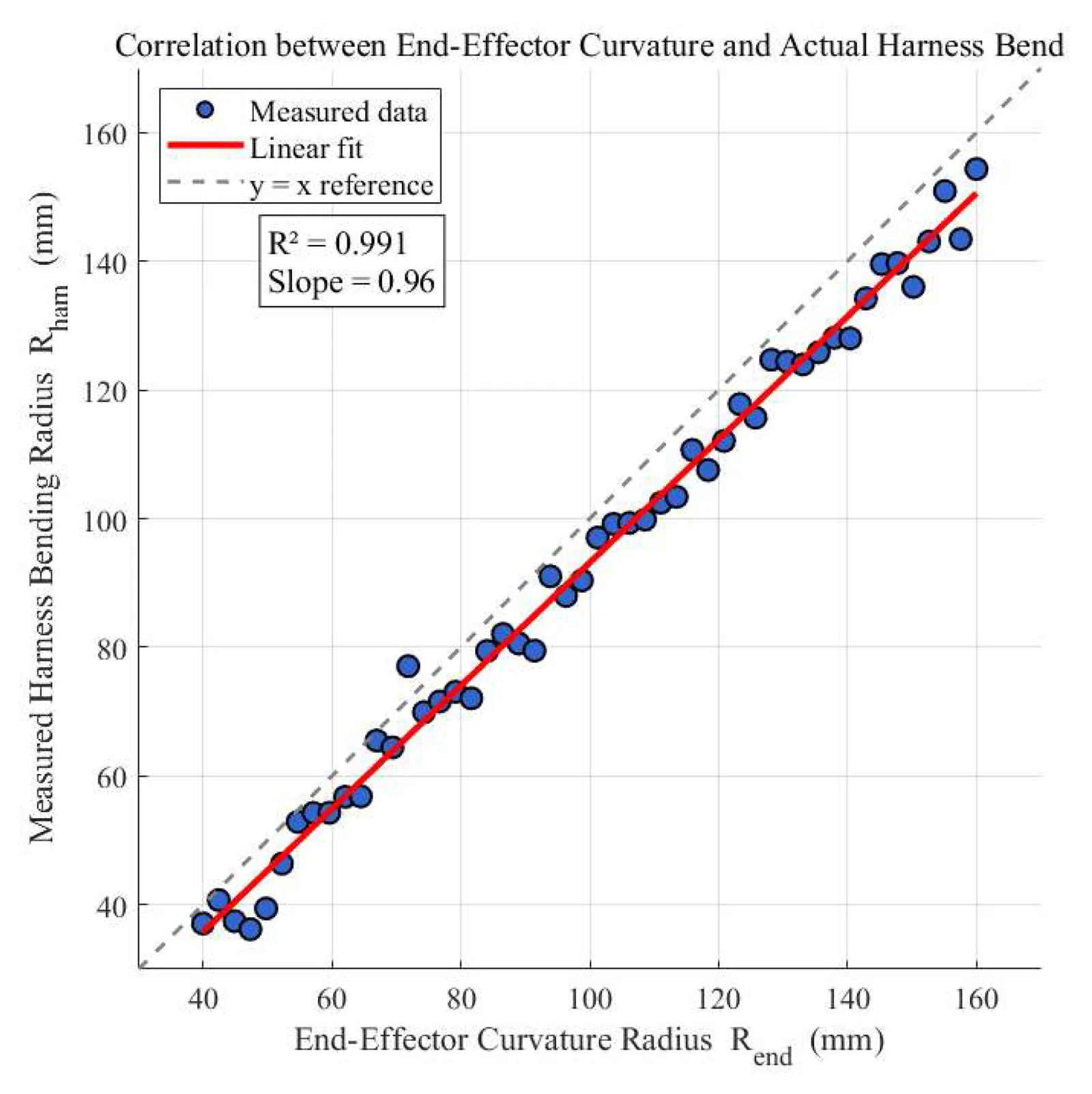
Correlation between the end-effector curvature radius $R_{end}$ and the measured harness bending radius $R_{harn}$.

As shown in Figure 12, 50 paired samples show a strong linear correlation between $R_{end}$ and $R_{harn}$, with $R^{2}=0.991$ and a fitted slope of 0.96. This indicates that, under the tested wire diameter, guiding-tool geometry, and routing condition, the end-effector curvature radius can reasonably reflect the variation trend of the actual harness bending radius. Therefore, $f_{3}$ is used in this study as an experimentally validated geometric bending-risk proxy rather than a complete flexible-body deformation model.

To further evaluate actual bending safety, the measured harness bending radius was compared with the required minimum bending radius $R_{min}=60\;\mathrm{mm}$. The violation number $N_{viol}$ is defined as the number of samples satisfying $R_{harn}<R_{min}$, and the bending-safety pass rate is calculated from 50 valid samples for each method.

As shown in Table 7, MOPSO and NSGA-II still produce several samples below the required bending-radius threshold. In contrast, the proposed method achieves $R_{harn,min}=64.8\;\mathrm{mm}$, which is higher than $R_{min}=60\;\mathrm{mm}$, with no bending-radius violation and no controller alarm during repeated physical execution. These results provide direct experimental evidence that the proposed bending-risk proxy can reduce actual local over-bending risk in robotic aircraft harness routing.

### 5.7. Discussion

The main methodological contribution of this study is the coupling of conventional robot-motion performance indicators with a process-risk representation specific to aircraft wire-harness routing. A trajectory satisfying joint limits and geometric collision constraints may still be unsuitable for physical production if it produces excessive local curvature near fixture-board pins. The proposed pin-neighborhood weighted bending-safety objective, therefore, extends the optimization target from geometric executability to process-safe executability. Moreover, the vision-based experiment shows that the end-effector curvature radius is strongly correlated with the measured harness bending radius ($R^{2}=0.991$), supporting the physical relevance of the proposed geometric proxy under the tested conditions.

The physical experiments further clarify the engineering significance of the method. Although MOPSO provides a marginally shorter execution time, the proposed method reduces normalized joint jerk by 35.8% and the bending-safety proxy by 77.0%, while completing all ten repeated trials without controller alarms. Vision-based measurements also show no bending-radius violation among the tested samples. For aircraft wire-harness manufacturing, where local over-bending, insulation damage, and inconsistent forming quality are unacceptable, the improvement in motion quality and process reliability is more important than a small reduction in cycle time. The successful execution of layouts containing up to 18 key points and four branches further indicates that the framework is not restricted to the representative six-point case.

Nevertheless, the proposed bending-safety metric remains a geometric proxy rather than a complete flexible-body deformation model. Its relationship with the actual harness shape may vary with material stiffness, diameter, tension, contact friction, and end-guiding-tool geometry. In addition, the current constraints are mainly verified using discrete trajectory samples, and the optimization remains primarily offline. Future work will therefore incorporate flexible-body dynamics, continuous-time constraint verification, and force/vision feedback for closed-loop trajectory correction under actual routing disturbances.

## 6. Conclusions

This study developed a process-safety-aware multi-objective trajectory optimization method for a seven-axis aircraft wire-harness routing robot. Its principal contribution is the integration of a pin-neighborhood weighted bending-safety proxy with execution time, normalized joint jerk, and coupled robot–process constraints, together with a constraint-guided adaptive DE-NSGA-II solver for the resulting narrow feasible region. Physical experiments showed that the proposed method reduced normalized jerk by 35.8% and the bending-safety proxy by 77.0% relative to MOPSO while completing all ten repeated trials without controller alarms. Vision-based measurements yielded a strong correlation ($R^{2}=0.991$) between the proxy and the measured harness bending radius, and additional layouts with up to 18 key points and four branches demonstrated the applicability of the method to more complex routing configurations. These results confirm that the proposed method provides a practical balance between routing efficiency and process reliability. Future work will incorporate flexible-body dynamics, continuous-time constraint verification, and force/vision feedback for closed-loop trajectory correction.

## Figures and Tables

**Table 1:** D-H parameters for the wiring robot.

Link i	*α_i_*/(rad)	*d_i_*/(mm)	*a_i_*/(mm)	*θ_i_*/(°)	Range
0	0	$d_{7}$	0	0	0–4400 mm
1	π2	200	0	$\theta_{1}$	−360°~+360°
2	0	0	250	$\theta_{2}$	−135°~+135°
3	0	0	300	$\theta_{3}$	−153°~+153°
4	−π2	150	0	$\theta_{4}$	−360°~+360°
5	π2	0	0	$\theta_{5}$	−180°~+180°
6	−π2	100	0	$\theta_{6}$	−360°~+360°

**Table 2:** Simulation settings and algorithm parameters.

Parameter	Value
Number of key path points	6
Effective board length/routing width	5000 mm/800 mm
Minimum bending radius $R_{min}$	60 mm
Board clearance $h_{min}$	30 mm
Non-target pin safety distance $d_{pin}$	40 mm
Pin-passing tolerance $\varepsilon_{pin}$	3 mm
Degree/control points/sampling points	5/12/1000
Initial time/search range	20 s/12–24 s
Seventh-axis/rotational-joint control-point offset	±80 mm/±8°
Population size/maximum generations/independent runs	200/200/20
Crossover probability/mutation probability	0.9/1/D
Inertia weight/learning factor/archive size	0.9–0.4/1.5/200
DE scaling factor F/crossover probability CR	0.4–0.8/0.6–0.95
Feasible-rate threshold/elite-guidance probability $p_{best}$	0.6/0.3

**Table 3:** Performance comparison and analysis of ablation experiments.

Algorithm Variant	HV	IGD	Spread	Feasibility Rate
Full proposed	0.84	0.04	0.30	97%
*w*/*o* CPO	0.78	0.06	0.35	88%
*w*/*o* FRD-DE	0.80	0.055	0.32	93%
*w*/*o* HR	0.70	0.10	0.45	72%
*w*/*o* FPS	0.75	0.075	0.38	85%

**Table 4:** Joint-space path-point values of the robot.

Path Point	Seventh-Axis Displacement (mm)	$\theta_{1}$ (deg)	$\theta_{2}$ (deg)	$\theta_{3}$ (deg)	$\theta_{4}$ (deg)	$\theta_{5}$ (deg)	$\theta_{6}$ (deg)
$P_{0}$	0.00	33.54	−55.12	120.45	0.00	24.67	33.54
$P_{1}$	220.50	28.41	−48.20	110.30	5.20	27.90	23.21
$P_{2}$	655.80	25.10	−42.50	102.10	8.40	30.40	16.70
$P_{3}$	1480.20	26.80	−43.10	103.50	6.10	29.60	20.70
$P_{4}$	2300.50	27.50	−44.20	105.80	3.20	28.40	24.30
$P_{5}$	2500.00	29.15	−49.80	112.40	0.00	27.40	29.15

**Table 6:** Summary of additional physical validation cases (all cases performed on the same 5000 mm × 800 mm fixture board).

Case	No. of Key Points	No. of Branches	Pin Arrangement	Execution Result	Controller Alarms	Success Rate
Case 1	6	1	Uniform	Passed	None	10/10
Case 2	10	2	Localized dense	Passed	None	9/10
Case 3	12	3	Uniform	Passed	None	10/10
Case 4	8	1	Non-uniform	Passed	None	10/10
Case 5	18	4	Mixed dense	Passed	None	9/10

**Table 7:** Vision-based validation of actual harness bending safety.

Method	$R_{harn,min}$/mm	$R_{harn,avg}$/mm	$N_{viol}$	Bending Safety Pass Rate	Controller Alarms
MOPSO	52.4	69.8	11/50	78%	2/10
NSGA-II	57.6	76.5	4/50	92%	1/10
Proposed	64.8	86.2	0/50	100%	0/10

## Data Availability

The data that support the findings of this study are available from the corresponding author.
